# The impact of subjective recognition experiences on recognition heuristic use: A multinomial processing tree approach

**DOI:** 10.3758/s13423-014-0587-4

**Published:** 2014-03-18

**Authors:** Marta Castela, David Kellen, Edgar Erdfelder, Benjamin E. Hilbig

**Affiliations:** 1 Department of Psychology, School of Social Sciences, Universität Mannheim, Schloss Ehrenhof Ost, 68161 Mannheim, Germany; 2 Albert-Ludwigs-Universität Freiburg, Freiburg im Breisgau, Germany; 3 Department of Psychology, School of Social Sciences, Universität Mannheim, 68161 Mannheim, Germany

**Keywords:** Recognition heuristic, Memory-state heuristic, Recognition memory, Decision making, Multinomial processing tree models

## Abstract

The recognition heuristic (RH) theory states that, in comparative judgments (e.g., Which of two cities has more inhabitants?), individuals infer that recognized objects score higher on the criterion (e.g., population) than unrecognized objects. Indeed, it has often been shown that recognized options are judged to outscore unrecognized ones (e.g., recognized cities are judged as larger than unrecognized ones), although different accounts of this general finding have been proposed. According to the RH theory, this pattern occurs because the binary recognition judgment determines the inference and no other information will reverse this. An alternative account posits that recognized objects are chosen because knowledge beyond mere recognition typically points to the recognized object. A third account can be derived from the memory-state heuristic framework. According to this framework, underlying memory states of objects (rather than recognition judgments) determine the extent of RH use: When two objects are compared, the one associated with a “higher” memory state is preferred, and reliance on recognition increases with the “distance” between their memory states. The three accounts make different predictions about the impact of subjective recognition experiences—whether an object is merely recognized or recognized with further knowledge—on RH use. We estimated RH use for different recognition experiences across 16 published data sets, using a multinomial processing tree model. Results supported the memory-state heuristic in showing that RH use increases when recognition is accompanied by further knowledge.

**Electronic supplementary material:**

The online version of this article (doi:10.3758/s13423-014-0587-4) contains supplementary material, which is available to authorized users.

The recognition heuristic (RH) for comparative judgments is among the simplest heuristics proposed by Goldstein and Gigerenzer (2002) within their program of the “adaptive toolbox”—metaphorically standing for decision makers’ repertoire of judgment and choice strategies. For pairwise comparisons, the RH can be stated as follows: “If one of two objects is recognized and the other is not, then infer that the recognized object has the higher value with respect to the criterion” (Goldstein & Gigerenzer, 2002, p. 76). For the RH to be applied, the following preconditions have been proposed: (1) recognition is a valid cue strongly correlated with the criterion; (2) further cues are not openly available; (3) recognition stems from natural encounters in the world (Gigerenzer & Goldstein, 2011).

The typical paradigm for investigating the RH consists of a comparison task in which participants see pairs of objects and must infer, for each pair, which object has a higher value on a *criterion dimension*. The most common example is the *city-size* task in which participants decide which of two cities has the larger population. Additionally, participants engage in a recognition task for each object. That is, they state for each object whether they recognize it or not. On the basis of this information, three types of object pairs can be defined: *recognition pairs* (one object is recognized and the other is not), *knowledge pairs* (both objects are recognized), and *guessing pairs* (neither of the objects is recognized). In some experiments, the recognition task additionally asks participants to state whether they merely recognized the name of the object or whether they have further knowledge about it (e.g., Hilbig & Pohl, 2009). However, despite this distinction of *recognition experiences*, participants’ judgments are usually simply analyzed as recognized versus unrecognized (some exceptions are Hilbig & Pohl, 2009; Hilbig, Pohl, & Bröder, 2009).

Several studies showed that recognized objects are chosen more often than unrecognized ones in recognition pairs (for reviews, see Gigerenzer & Goldstein, 2011; Pachur, Todd, Gigerenzer, Schooler, & Goldstein, 2011). However, choosing the recognized object does not necessarily involve use of the RH. Whereas the latter implies that recognition alone determined the choice, the former can occur either from consideration of recognition alone or in combination with further knowledge about the recognized object (which will typically be in line with the recognition cue). In this sense, different accounts have been proposed for the observable tendency to choose the recognized object. According to the original RH theory, the recognized object is chosen more often because “if one object is recognized and the other one is not, then the inference is determined; no other information about the recognized object is searched for and, therefore, no other information can reverse the choice determined by recognition” (Goldstein & Gigerenzer, 2002, p. 82). We will refer to this account as the *invariance account*.

An alternative account, which we will designate as the *inhibition account*, presumes that the recognition cue can be overruled by further knowledge. Specifically, the recognized object is chosen more often not for being recognized per se, but because further information about this object leads to the same choice. This account is corroborated by several studies showing that further knowledge affects choices in recognition pairs (e.g., Bröder & Eichler, 2006; Hilbig & Richter, 2011; Newell & Fernandez, 2006). For example, people are more likely to infer that a recognized city is more populous than an unrecognized one if they know that the recognized city has a major league soccer team (Newell & Fernandez, 2006). Naturally, further knowledge can also result in the choice of the unrecognized object when the available information indicates that the recognized object is small. Nevertheless, since nothing is known (and little can be inferred) about unrecognized objects, knowledge will typically support choice of recognized objects.

A third account is given by the *memory-state heuristic* (MSH; Erdfelder, Küpper-Tetzel, & Mattern, 2011). The MSH presumes that individuals tend to choose the object that reaches a “higher” memory state—that is, a higher level of memory strength. Because criterion values are typically strongly correlated with memory strengths (Erdfelder et al., 2011), MSH use will often result in correct inferences. In line with the two-high-threshold model of recognition (e.g., Kellen, Klauer, & Bröder, 2013), the MSH assumes that objects are in one of three memory states: *recognition certainty*, *uncertainty*, or *rejection certainty*. Objects with memory strengths exceeding a *recognition threshold* are in the recognition certainty state and are judged as recognized. If the memory strength falls below this recognition threshold but is still larger than a *rejection threshold*, an object is in the uncertainty state, and the recognition judgment is determined by guessing. Finally, if the memory strength falls below the rejection threshold, an object is in the rejection certainty state and is judged as unrecognized. According to the MSH, reliance on recognition should increase with the “distance” between memory states of the to-be-compared objects. Specifically, if one object is in the recognition certainty state and the other in the rejection certainty state, reliance on recognition should be highest.

## Beyond binary recognition judgments: New predictions

As was previously mentioned, the majority of studies investigating the RH have relied on binary recognition judgments, ignoring the reported subjective recognition experiences. However, when distinguishing between nonrecognition (*U*), mere recognition (*mR*), and recognition with further knowledge (*R* ^+^) judgments, it can be seen that the different accounts make distinct predictions.

According to the invariance account, RH use should not vary with the composition of the recognition pairs (i.e., pairs judged *R* ^+^–*U* vs. *mR–U*), because only the binary recognition judgment determines choices and the distinction between *R* ^+^ and *mR* should not matter. In contrast, the inhibition account predicts that RH use will be less frequent for *R* ^+^–*U* pairs than for *mR–U* pairs, since the availability of knowledge should lead to integration of this knowledge and, by implication, decrease reliance on the RH. The MSH account makes the opposite prediction; that is, RH use should be more frequent for *R* ^+^–*U* than for *mR–U* pairs, because it is more likely that the recognized object in the former pair is in the recognition certainty state than that the recognized object in the latter pair is. Note that this prediction assumes only that reported recognition experiences (*R* ^+^ vs. *mR*) and underlying memory states (recognition certainty vs. uncertainty) are positively correlated. It does not require that all *R* ^+^ objects be in the recognition certainty state. To derive the MSH prediction, it suffices to assume that *R* ^+^ objects more likely originate from recognition certainty than *mR* objects do.

The MSH account makes an interesting additional prediction. Specifically, the availability of further knowledge should be used as a cue in *R* ^+^–*mR* knowledge pairs as well, leading to the *R* ^+^ object being judged as having a higher criterion value (e.g., being judged as the more populous city). Again, this prediction emerges from the fact that *R* ^+^ objects are more likely in a recognition certainty state than *mR* objects. The other two accounts make no such prediction, since they predict that choices for knowledge pairs will be based on retrieved knowledge only.

Finally, predictions regarding the ecological validity of the different recognition experiences can also be made. According to the MSH account, objects in the recognition certainty state should have higher criterion values than objects in the uncertainty state (Erdfelder et al., 2011). Thus, the MSH predicts that the probability of the recognized object having the larger criterion value should be greater for *R* ^+^–*U* than for *mR–U* pairs. The invariance account predicts no such difference, because *R* ^+^ and *mR* objects are treated as equivalent if compared with unrecognized objects.

The evaluation of the above-described predictions requires the ability to disentangle the relative contributions of RH use and reliance on further knowledge. The *r-model* proposed by Hilbig, Erdfelder, and Pohl (2010) provides such a measure of RH use (via parameter *r*), while also taking into account the contribution of further knowledge. However, this model does not distinguish between different types of recognition experiences. In the next section, we first present the r-model and then propose an extension, the r*-model, that incorporates different recognition experiences.

## From the r-model to the r*-model

The r-model belongs to the class of multinomial processing tree models (Batchelder & Riefer, 1999; Erdfelder et al., 2009). This class of models assumes that the observed categorical responses are produced by a set of discrete mental states. The probability of each state being entered is determined by the probability of certain cognitive processes taking place or not. The models provide estimates for the probability of each of these processes taking place, producing a characterization of categorical data in terms of latent cognitive processes. Multinomial processing tree models are usually depicted as trees, with each branching presenting the occurrence (or not) of cognitive processes and the terminal nodes representing the observed categorical responses.

**Fig. 1:**
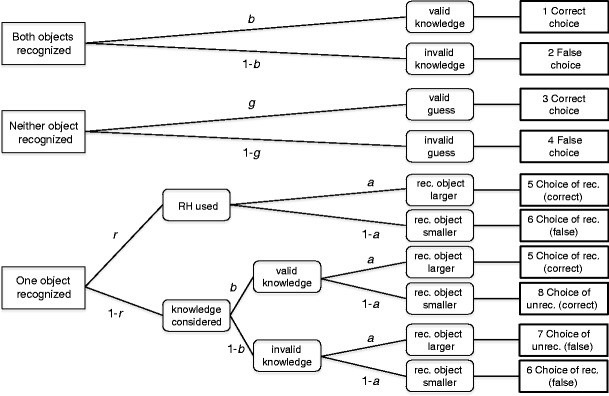
Parameter *r* denotes the probability of applying the recognition heuristic as originally proposed—that is, by ignoring any knowledge beyond recognition. *a* = recognition validity (probability of the recognized object representing the correct choice when paired with an unrecognized object); *b* = probability of valid knowledge; *g* = probability of a correct guess; rec. = R = recognized; unrec. = U = unrecognized

The r-model (Hilbig, Erdfelder, & Pohl, 2010) models data from a two-alternative forced choice comparison task and a yes–no recognition task. The recognition judgments are used to categorize the pairs into knowledge, recognition, or guessing cases, defining the three trees of the model (see Fig. 1). They lead to eight outcome categories that are described by four parameters: *r*, the probability of applying the recognition heuristic; *a*, the probability of recognition being a valid cue; *b*, the probability of valid knowledge; and *g*, the probability of a correct guess. While both the knowledge and guessing trees are defined by a single parameter that accounts for accuracy (*b* and *g*, respectively), the recognition tree is slightly more complex. If the RH is used (with probability *r*), accuracy depends on recognition validity; with probability *a*, the inference will be correct; and with probability 1 − *a*, it will be false.^1^ If further knowledge or any other judgment strategy is used, the RH is not applied (with probability 1 − *r*), and accuracy depends on (knowledge) validity. With probability *b*, the answer is correct, and with probability 1 – *b*, it is false. Again, the choice of either the recognized or the unrecognized object will depend on the recognition validity (but see footnote 1).

**Fig. 2:**
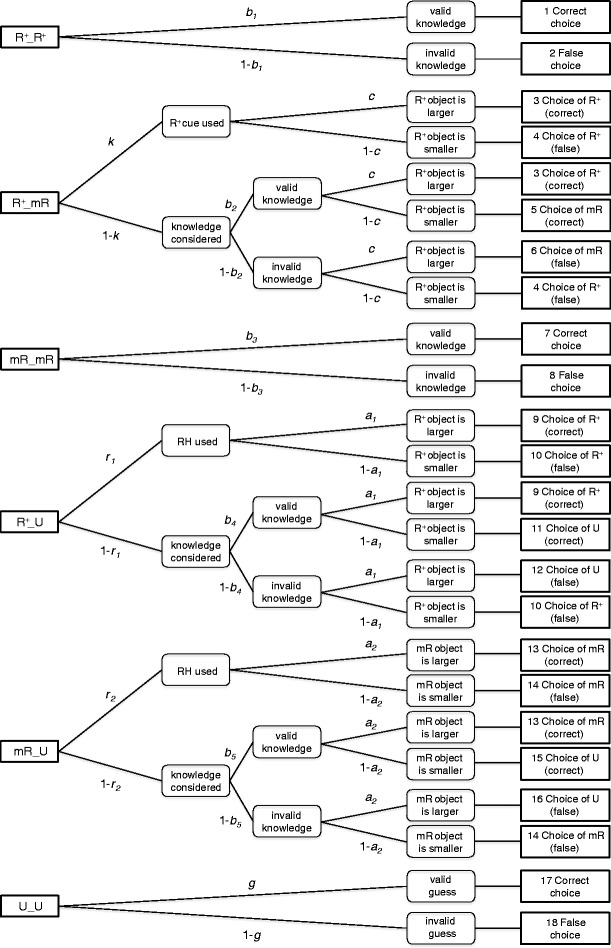
Tree representation of the r*-model. *R* ^+^, object recognized with further knowledge; *mR*, object merely recognized; *U*, object unrecognized; *b*_1_, *b*_2_, and *b*_3_, knowledge validity parameters; *k*, probability of using the further knowledge cue; *c*, validity of choosing the *R* ^+^ object (probability that it represents the correct choice) in *R* ^+^–*mR* pairs; *r*_1_, probability of applying the recognition heuristic (RH) in pairs for which the recognized object received an *R* ^+^ judgment; *a*_1_, recognition validity (probability of the recognized object representing the correct choice) in pairs for which the recognized object received an *R* ^+^ judgment; *r*_2_, probability of applying the RH in pairs for which the recognized object received an *mR* judgment; *a*_2_, recognition validity (probability of the recognized object representing the correct choice) in pairs for which the recognized object received an *mR* judgment; *g*, probability of a valid guess

To investigate whether use of the RH varies between recognition pairs in which the recognized object is judged as either *R* ^+^ or *mR*, we extended the r-model to the r*-model (see Fig. 2). The r*-model consists of six trees with 18 outcome categories in total. Because the category probabilities must sum up to one for each tree, only 12 of the 18 probabilities are free to vary. These category probabilities are represented by 10 parameters, resulting in a testable model with 12 − 10 = 2 degrees of freedom. The r*-model comprises three trees for knowledge cases, two trees for recognition cases, and one guessing tree. The three knowledge trees refer to (1) *R* ^+^–*R* ^+^ pairs, (2) *R* ^+^–*mR* pairs, and (3) *mR–mR* pairs. It could be argued that this is not a knowledge tree, since, according to the participant’s judgments, there is no knowledge available. Nevertheless, we refer to the parameter that accounts for accuracy in these pairs as a knowledge parameter, but more for reasons of consistency and simplicity than due to a strong claim about the availability of valid knowledge for these cases. The two recognition trees correspond to simple duplications of the original recognition tree in the r-model (each with its own set of *r* and *b* parameters), accounting both for *R* ^+^–*U* and *mR–U* pairs. Finally, as in the r-model, the guessing tree includes pairs of two unrecognized objects (*U–U*).

As can be seen in Fig. 2, in the *R* ^+^–*mR* knowledge tree, we assume that the distinction between merely recognized objects (*mR*) and recognized objects with further knowledge (*R* ^+^) can be used as a simple cue. In other words, irrespective of the retrieved knowledge, the *R* ^+^ object would be preferred over the *mR* object (as measured by parameter *k*). If participants use this strategy (as predicted by the MSH), a correct answer depends on the *R* ^+^ cue’s validity (as measured by parameter *c*)—that is, on the proportion of times the object with the higher criterion value is the one judged as *R* ^+^. However, if this strategy is not used, participants rely on the knowledge they possess, and a correct answer will depend on the validity of knowledge (as measured by parameter *b*_2_). Choice of the *R* ^+^ or the *mR* object will again depend on parameter *c*.

### Model-based hypothesis testing

The hypotheses discussed previously can be represented by parameter restrictions in the r*-model:

$$ \mathrm{invariance}\kern0.5em \mathrm{account}:{r}_1={r}_2,\kern1em {a}_1={a}_2,\mathrm{inhibition}\kern0.5em \mathrm{account}:{r}_1<{r}_2,\mathrm{MSH}:{r}_1>{r}_2,\kern1em {a}_1>{a}_2. $$

In addition to these restrictions, the MSH predicts that people use the strategy modeled by parameter *k*. Therefore, the MSH predicts that the restriction *k* = 0 should produce gross misfits.

The suitability of the different parameter restrictions can be compared by evaluating the relative performance of the models instantiating them. A model selection analysis will allow us to assess which hypotheses are corroborated by the data and which are rejected. Model selection requires a weighting between the ability of each model to account for the observed data and the ability of each model to account for data in general (model complexity or flexibility), since more flexible models provide a better fit to data a priori. The goal is to find the model with the best trade-off between fit and flexibility (see Vandekerckhove, Matzke, & Wagenmakers, in press).

One prominent approach in model selection is based on the minimum description length principle (MDL; Kellen et al., 2013). According to the MDL approach, both models and data are understood as codes that can be compressed. The goal of MDL is to assess models in terms of their ability to compress data. The greater the compression, the better the account of the underlying regularities that are present in the data. One of the indices emerging from the MDL principle is the Fisher information approximation (FIA), which combines a model’s goodness of fit with model flexibility penalties:1


The first summand of FIA corresponds to the (minus) maximum log-likelihood of observed data *x* in a particular experiment, quantifying model ℳ’s fit, and the second and third summands correspond to the model penalties. The second summand takes the number of parameters *p* and sample size *N* into account. The third summand accounts for the flexibility of the model due to its functional form by integrating over the determinant of the expected Fisher information matrix *I*(*θ*). FIA differences larger than 1.1 already represent substantial evidence in favor of the winning model (Kellen et al., 2013).

### Analysis of data sets

The r*-model requires responses discriminating between objects that were unrecognized, merely recognized, and recognized with further knowledge. Sixteen previously published data sets fulfilled this requirement (Hilbig, Erdfelder, & Pohl, 2010, 2011, 2012; Hilbig & Pohl, 2008, 2009; Hilbig et al., 2009; Hilbig, Scholl, & Pohl, 2010). The choice task used in all data sets was the city-size task. Table 1 provides a description of each data set (additional details can be found in the Supplemental Material). FIA values and parameter estimates were calculated using the MPTinR package (Singmann & Kellen, 2013). Following Hilbig, Erdfelder, Pohl (2010), the baseline restrictions *b*_1_ = *b*_4_ and *b*_3_ = *b*_5_ were imposed on the model.

**Table 1:** Data sets

Data Set	Origin	Materials	*N*
1	Hilbig & Pohl, 2009, Experiment 1	20 largest Swiss cities	4,560
2	Hilbig & Pohl, 2009, Experiment 2	17 random world cities	9,969^∗^
3	Hilbig & Pohl, 2009, Experiment 3	14 largest Swiss cities	6,188
4	Hilbig & Pohl, 2008, Experiment 5	11 random world cities	5,776^∗^
5	Hilbig, Erdfelder, & Pohl, 2011	14 Polish and 14 Austrian cities	12,012
6	Hilbig, Pohl, & Bröder, 2009	14 largest Belgian cities	7,358^∗^
7	Hilbig, Erdfelder, & Pohl, 2010 (6a)	17 random world cities	2,312
8	Hilbig, Erdfelder, & Pohl, 2010 (6b)	17 random world cities	2,584
9	Hilbig, Erdfelder, & Pohl, 2010 (7a)	14 largest Italian cities	1,183
10	Hilbig, Scholl, & Pohl, 2010, Experiment 1a	16 largest Canadian cities	1,320
11	Hilbig, Scholl, & Pohl, 2010, Experiment 1b	16 largest Canadian cities	960
12	Hilbig, Scholl, & Pohl, 2010, Experiment 2a	16 largest Canadian cities	2,400
13	Hilbig, Scholl, & Pohl, 2010, Experiment 2b	16 largest Canadian cities	2,040
14	Hilbig, Erdfelder, & Pohl, 2012, Experiment 1a	18 random world cities	3,672
15	Hilbig, Erdfelder, & Pohl, 2012, Experiment 1b	18 random world cities	3,213
16	Hilbig, Erdfelder, & Pohl, 2012, Experiment 1c	18 random World cities	3,672

*Note.* The sample size corresponds to the aggregate level: total number of trials multiplied by number of participants. For the data sets marked with an *, the total *N* does not match what was reported in the published articles. This is due to missing values in variables required for the analysis.

The baseline model performed well in describing the data (see Table 2). For 12 of the 16 data sets, it fitted the data according to the standard *G* ^2^ goodness-of-fit test using *α* = .05 as a criterion of significance. For 4 of the 16 data sets (data sets 5, 13, 15, and 16), there was misfit at this level of significance. However, these misfits did not exceed the critical *G* ^2^ values obtained in compromise power analysis (i.e., balancing of type I and type II error probabilities) given an effect size of *ω* = 0.1 under *H*_1_ (see Erdfelder, 1984; Faul, Erdfelder, Lang, & Buchner, 2007).

**Table 2:** Model fit and maximum likelihood parameter estimates

Data Set	*G* ^2^	*p*-value	*b*_1_	*b*_2_	*b*_3_	*k*	*c*	*g*	*r*_1_	*r*_2_	*a*_1_	*a*_2_
1	4.85	.09	.75 (.02)	.85 (.02)	.68 (.02)	.37 (.05)	.80 (.02)	.52 (.02)	.77 (.03)	.63 (.03)	.93 (.01)	.79 (.01)
2	2.44	.30	.70 (.01)	.73 (.02)	.62 (.02)	.46 (.03)	.70 (.01)	.54 (.01)	.73 (.01)	.45 (.03)	.82 (.01)	.74 (.01)
3	3.67	.16	.74 (.01)	.78 (.02)	.64 (.02)	.42 (.03)	.70 (.01)	.56 (.02)	.84 (.02)	.67 (.02)	.82 (.01)	.73 (.01)
4	0.94	.62	.65 (.01)	.67 (.03)	.52 (.02)	.60 (.02)	.48 (.02)	.53 (.02)	.70 (.02)	.49 (.03)	.57 (.01)	.62 (.01)
5	8.00	.02	.66 (.02)	.69 (.02)	.63 (.01)	.50 (.02)	.65 (.01)	.53 (.01)	.82 (.01)	.70 (.02)	.86 (.01)	.81 (.01)
6	5.08	.08	.69 (.02)	.71 (.04)	.64 (.02)	.61 (.04)	.78 (.02)	.57 (.01)	.82 (.02)	.52 (.02)	.94 (.01)	.78 (.01)
7	3.82	.15	.64 (.03)	.84 (.04)	.66 (.03)	.50 (.06)	.72 (.03)	.52 (.02)	.74 (.03)	.63 (.04)	.79 (.02)	.70 (.02)
8	1.34	.51	.63 (.02)	.63 (.04)	.61 (.04)	.50 (.05)	.58 (.03)	.51 (.02)	.84 (.02)	.75 (.04)	.79 (.01)	.77 (.02)
9	0.99	.61	.71 (.04)	.81 (.05)	.53 (.05)	.41 (.11)	.86 (.03)	.50 (.03)	.75 (.05)	.57 (.06)	.94 (.01)	.69 (.03)
10	1.42	.49	.52 (.08)	.65 (.09)	.51 (.04)	.64 (.08)	.67 (.04)	.59 (.02)	.98 (.01)	.67 (.04)	.82 (.02)	.74 (.02)
11	0.03	.98	.58 (.06)	.72 (.10)	.58 (.04)	.67 (.09)	.75 (.04)	.54 (.03)	.95 (.02)	.50 (.06)	.82 (.02)	.70 (.03)
12	2.38	.30	.62 (.03)	.67 (.04)	.56 (.03)	.40 (.06)	.62 (.03)	.53 (.02)	.77 (.03)	.52 (.04)	.80 (.02)	.68 (.02)
13	6.09	.05	.63 (.03)	.84 (.05)	.62 (.04)	.60 (.07)	.75 (.03)	.53 (.02)	.85 (.02)	.56 (.05)	.78 (.02)	.68 (.02)
14	3.17	.20	.66 (.01)	.74 (.03)	.67 (.02)	.30 (.04)	.56 (.02)	.45 (.02)	.56 (.03)	.42 (.04)	.59 (.02)	.59 (.02)
15	6.48	.04	.68 (.01)	.75 (.03)	.64 (.03)	.39 (.04)	.55 (.02)	.50 (.02)	.58 (.03)	.41 (.05)	.64 (.01)	.57 (.03)
16	8.07	.02	.64 (.02)	.62 (.03)	.64 (.02)	.13 (.05)	.53 (.02)	.46 (.02)	.69 (.02)	.63 (.03)	.57 (.02)	.56 (.02)
Mean	3.67	–	.66	.73	.61	.47	.67	.52	.77	.57	.78	.70

*Note.* Standard errors in parentheses

The results reported in Table 3 show that for the majority of the data sets (12 out of 16), the FIA metric prefers the model imposing the full set of MSH restrictions, *r*_1_ > *r*_2_ and *a*_1_ > *a*_2_, and provides support for *k* > 0. These results are corroborated by the parameter estimates obtained with the unrestricted model, which are almost invariably consistent with these parameter restrictions (see Table 2).^2^

**Table 3:** Model-Selection Results: FIA indices for different versions of the r*-model applied to 16 data sets

	Parameter Restrictions								
Data Set	baseline	*r*_1_ = *r*_2_	*r*_1_ = *r*_2_ *a*_1_ = *a*_2_	*r*_1_ = *r*_2_ *a*_1_ = *a*_2_ *k* = 0	*r*_1_ ≤ *r*_2_	*r*_1_ ≤ *r*_2_ *k* = 0	*r*_1_ ≥ *r*_2_ *a*_1_ ≥ *a*_2_	*r*_1_ ≥ *r*_2_ *a*_1_ = *a*_2_	*r*_1_ ≥ *r*_2_ *a*_1_ ≥ *a*_2_ *k* = 0
1	34.80	37.13	83.09	118.34	39.01	74.25	**33.41**	80.09	68.65
2	37.29	81.21	95.15	224.39	83.38	212.63	**35.92**	50.57	165.17
3	36.17	50.02	60.99	171.04	52.02	162.07	**34.79**	46.47	144.84
4	34.46	54.29	53.68	255.12	56.28	257.72	35.95	**33.18**	237.39
5	41.08	52.36	61.89	240.86	54.72	233.69	**39.68**	49.94	218.66
6	36.84	65.42	155.66	270.67	67.49	182.49	**35.45**	126.41	150.45
7	30.90	31.21	34.04	73.67	32.73	72.36	**29.50**	33.05	69.14
8	30.03	30.25	27.61	69.10	31.78	73.26	28.65	**26.74**	70.14
9	25.85	26.41	53.08	60.61	27.52	35.05	**24.48**	51.89	32.02
10	26.20	46.46	46.50	70.48	47.68	71.66	**24.79**	25.55	48.78
11	24.40	46.34	48.46	68.23	47.45	67.21	**23.02**	25.85	42.79
12	30.37	40.81	49.58	72.69	42.38	65.48	**28.98**	38.48	52.09
13	30.79	46.14	48.35	80.25	47.55	79.45	**29.43**	32.36	61.32
14	32.81	35.64	32.44	56.79	37.35	61.70	31.47	**28.98**	55.82
15	33.55	36.69	36.18	72.52	38.28	74.62	**32.19**	32.38	68.52
16	35.46	34.27	**31.27**	34.09	36.08	38.89	34.08	31.79	36.90
Total	521.00	714.65	917.97	1,938.85	741.70	1,762.53	**501.79**	713.73	1,522.68

*Note.* FIA indices of the winning model for each data set are set in boldface type. Following Hilbig, Erdfelder, Pohl (2010), all models have the restriction *b*_1_ = *b*_4_ and *b*_3_ = *b*_5_. The baseline model had no further restrictions. Extending the set of candidate models by including models without these restrictions does not change the model selection results

Three data sets (4, 7, and 14) were better accounted for by a model imposing the restrictions *r*_1_ > *r*_2_ and *a*_1_ = *a*_2_. This departs from the MSH only in terms of the latter’s expected ecological validity, since the probability of the recognized object having the larger criterion value was not found to be reliably greater in *R* ^+^–*U* pairs than in *mR–U* pairs. Finally, data set 16 was better described by a model imposing the restrictions *r*_1_ = *r*_2_ and *a*_1_ = *a*_2_. As can be seen in the Supplemental Material, data set 16 corresponds to a condition in which speeded responses were collected. It is plausible that the retrieval of additional information from memory was impaired by this experimental constraint, leading to the use of fast, familiarity-based recognition judgments (e.g., Pachur & Hertwig, 2006).

## General discussion

We tested the predictions of three different accounts about the impact of subjective recognition experiences on RH use. Overall, we found a clear pattern that was predicted by the MSH and is inconsistent with both the invariance and the inhibition accounts. RH use is more frequent when the recognized object is judged as *R* ^+^ than when judged as *mR*. The MSH predictions about RH use for different recognition experiences rely on the assumption that objects judged as *R* ^+^ are more likely to have originated from a certainty state than objects judged as *mR*. Despite the plausibility of this assumption, future efforts should be placed on implementing a complete model that associates choice predictions to latent memory states that are themselves estimated from the data (Erdfelder et al., 2011; Pachur et al., 2011). This, however, implies the possibility of distinguishing whether an object (e.g., a city name) was experienced previously or not. One way to achieve this is by inducing recognition experimentally (see Bröder & Eichler, 2006), although it can be argued that this “artificial” recognition is beyond the domain of the RH (Gigerenzer & Goldstein, 2011).

In addition to the main hypotheses, we derived two other predictions from the MSH framework. The first prediction concerns a strategy that was not investigated before—namely, choosing the object judged as “recognized with further knowledge” (*R* ^+^) in a heterogeneous *R* ^+^–*mR* knowledge pair, irrespective of the retrieved knowledge. The observed use of this strategy suggests that participants are relying on a difference in memory states. The second prediction relates to the recognition validities in the two recognition trees. We observed that recognition validity was (in most data sets) higher in *R* ^+^–*U* than in *mR–U* recognition pairs. This shows that the MSH framework reflects the environmental structure better than does the invariance account. Both results reinforce the importance of memory states in adaptive decision making and, thus, the need to go beyond simple binary yes–no recognition judgments.

In sum, we found strong support for the MSH by testing the influence of recognition experiences on RH use. The inhibition account prediction that the availability of knowledge reduces RH use was not supported, and only in one data set (under time pressure conditions) did we find support for the invariance account prediction that RH use should not differ between recognition experiences. We believe that our work shows the importance of focusing on underlying memory processes when investigating memory-based probabilistic inferences and strategies such as the RH.

## Electronic supplementary material

Below is the link to the electronic supplementary material.

ESM 1 (ZIP 196 kb)

## Footnotes

1 The *a* parameter represents the proportion of recognition cases in which the recognized object has the larger criterion value. This parameter could be placed without loss of generality at the root of the tree or even removed implicitly via the use of two trees (for pairs in which the recognized item has the smaller or larger criterion value, respectively). We find the present parametrization the most convenient one for several (pragmatic) reasons.

2 The preference for this particular restricted model did not change when including equivalent candidate models that did not include the baseline restrictions *b*_1_ = *b*_4_ and *b*_3_ = *b*_5_. Moreover, the FIA-based results were corroborated by order-restricted significance tests on parameter restrictions (see the Supplemental Material).
