# Supplementary material for: The impact of subjective recognition experiences on recognition heuristic use: A multinomial processing tree approach
Source: Psychon Bull Rev. 2014 Mar 18;21(5):1131–8. doi: 10.3758/s13423-014-0587-4 (PMC4181781; doi:10.3758/s13423-014-0587-4)
Supplement: Supplementary file 1 — (ZIP 196 kb) [file 13423_2014_587_MOESM1_ESM.zip › Supplemental Material/description_datasets.pdf]

Data Sets

Data Sets 1 to 3 correspond to three experiments from Hilbig and Pohl (2009). In Experiment 1 (Data Set 1), the 20 largest Swiss cities were exhaustively paired (resulting in 190 comparisons) and 24 participants (17 female; 19 to 30 years old, $M = 23.9$, $SD = 3.1$) had to decide which of the two was larger. In Experiment 2 (Data Set 2), 74 (44 female; ages 18 to 46 years old, M = 22.2, SD = 5.3) participants had to perform 136 comparisons (95 trials were discarded due to missing data), resultant from an exhaustive pairing of a random selection of 17 cities (out of a list of the largest world cities). Finally, in Experiment 3 (Data Set 3) the material were the 14 largest Swiss cities (excluding Zürich) that were paired exhaustively, resulting in 91 paired comparisons, evaluated by 68 participants (56 female; ages 18 to 46, $M = 22.2$, $SD = 5.3$)

Data Set 4 corresponds to Experiment 5 in Hilbig and Pohl (2008). In this experiment, 106 participants (67 women; ages 19 to 57, $M = 24.4$, $SD = 6.7$) decided which of two cities was more populous throughout 55 pairs (composed of a sample of 11 cities out of a list of the 62 largest world cities). Fifty-four trials were discarded due to missing data.

In Data Set 5 − Hilbig, Erdfelder, and Pohl (2011) −, 66 (54 female; ages 18 to 46, $M = 22$, $SD = 5.3$) participants performed the judgment task for 91 pairs, consisting of 14 cities from two domains: Austria and Poland. Data was analyzed across domains, resulting in a total of 182 choices per participant.

In Data Set 6 − Hilbig, Pohl, and Bröder (2009) −, 81 (63 female; ages between 17 and 40, $M = 22.2$, $SD = 4.2$) participants performed the classical city-size task with the 14 largest Belgium cities (excluding the largest, Brussels) that were exhaustively paired resulting in 91 comparisons per participant (13 trials were discarded due to missing data).

Data Set 7 corresponds to Data Set 6 in Hilbig, Erdfelder, and Pohl (2010). The experiment included a manipulation aimed at validating parameter r of the r-model. For that purpose, the 36 (29 women; ages between 19 and 32, $M = 21.7$, $SD = 2.8$) participants were randomly assigned to one of the two conditions. The task included 17 randomly selected world cities resulting in 136 pairs. In the control condition (Data Set 7) there were no additional instructions. In the experimental group (Data Set 8) participants were asked to make use of the recognition cue whenever possible and to do so without necessarily considering further knowledge.

Data Set 9 − Data Set 7 from Hilbig, Erdfelder, and Pohl (2010) − was also analyzed. It corresponds to the control group of an experiment where 28 participants (20 women; ages between 18 and 33 years, $M = 21.9$, $SD = 3.9$) were randomly assigned to either a condition where they had to judge which of two Italian cities was more populous (Data Set 9) or an experimental group in which the same cities had to be compared regarding their height over sea level. For both, 14 cities

were exhaustively paired resulting in 91 comparisons. We did not include the experimental group in our analysis because recognition validity is very low for this domain, and participants do not use the recognition heuristic that often for it. Including this data would only create confusion, since there are no specific predictions for it and it is plausible that our model would not hold for this data.

We also analyzed two experiments from Hilbig, Scholl, and Pohl (2010). The only difference between the two is that in Experiment 1 participants were asked to select the more populous city, whereas in Experiment 2 they should choose the less populous city of the pair. In both experiments there were two groups with different instructions. Participants in the deliberation group (Data Set 10 and Data Set 12) were instructed to think carefully about each choice, while in the intuitive group (Data Set 11 and Data Set 13) they should decide spontaneously and according to gut feelings. The material corresponded to the 16 most populous Canadian cities paired exhaustively in 120 pairs. In Experiment 1 there were 19 participants (16 female; ages between 18 and 25 years, $M = 20.7$, $SD = 1.7$), and 37 participants in Experiment 2 (19 female; ages between 18 and 38 years, $M = 22.3$, $SD = 3.9$).

Finally, Data Sets 14 to 16 correspond to data from Experiment 1 in Hilbig, Erdfelder, and Pohl (2012). Sixty-nine participants (47 female; ages between 18 and 30 years, $M = 22$, $SD = 2.4$) made choices about 153 pairs composed of a selection of 18 of the largest world cities. There were three between-subjects conditions: baseline condition, participants were paid a flat fee independent of performance (Data Set 14); no-time-pressure condition, participants were paid according to their performance (Data Set 15); time-pressure condition, participants were again paid according to their performance but additionally they faced opportunity costs of time, meaning that being accurate would only be worthwhile if the choices were fast (Data Set 16).

References

Hilbig, B. E., Erdfelder, E., & Pohl, R. F. (2010). One-reason decision making unveiled: A measurement model of the recognition heuristic. *Journal of Experimental Psychology: Learning, Memory, and Cognition*, *36*(1), 123-134.

Hilbig, B. E., Erdfelder, E., & Pohl, R. F. (2011). Fluent, fast, and frugal? A formal model evaluation of the interplay between memory, fluency, and comparative judgments. *Journal of Experimental Psychology: Learning, Memory, and Cognition*, *37*(4), 827-839.

Hilbig, B. E., Erdfelder, E., & Pohl, R. F. (2012). A matter of time: Antecedents of one-reason decision making based on recognition. *Acta Psychologica*, *141*(1), 9–16.

Hilbig, B. E., & Pohl, R. F. (2008). Recognizing users of the recognition heuristic. *Zeitschrift für Experimentelle Psychologie / Experimental Psychology*, *55*(6), 394–401.

Hilbig, B. E., & Pohl, R. F. (2009). Ignorance-versus evidence-based decision making: A decision time analysis of the recognition heuristic. *Journal of Experimental Psychology: Learning, Memory, and Cognition*, *35*(5), 1296–1305.

Hilbig, B. E., Pohl, R. F., & Bröder, A. (2009). Criterion knowledge: A moderator of using the recognition heuristic? *Journal of Behavioral Decision Making*, *22*(5), 510–522.

Hilbig, B. E., Scholl, S. G., & Pohl, R. F. (2010). Think or blink − Is the recognition heuristic an intuitive strategy. *Judgment and Decision Making*, *5*(4), 300–309.
