# Supplementary material for: The impact of subjective recognition experiences on recognition heuristic use: A multinomial processing tree approach
Source: Psychon Bull Rev. 2014 Mar 18;21(5):1131–8. doi: 10.3758/s13423-014-0587-4 (PMC4181781; doi:10.3758/s13423-014-0587-4)
Supplement: Supplementary file 1 — (ZIP 196 kb) [file 13423_2014_587_MOESM1_ESM.zip › Supplemental Material/Results.pdf]

Order-restricted significance tests

Most of the hypotheses that we wish to test involve inequalities across parameters (e.g., $r_1 \geq r_2$) or parameters at the boundary of the parameter space (e.g., $k = 0$). In such cases the sampling distribution of the likelihood-ratio test statistic ΔG^2 no longer follows a χ^2 distribution with the appropriate number of degrees of freedom, but a $\overline{\chi}^2$ distribution, which consists of a *mixture* of χ^2 distributions with different number of degrees of freedom (Self & Liang, 1987; Shapiro, 1985; for an introduction see Iverson, 2006). For a single data set, the sampling distribution of the ΔG^2 (under the null hypothesis) resulting from the testing of a single inequality restriction is given by $\overline{\chi}^2$, with $\overline{\chi}^2 \sim \frac{1}{2}\chi_0^2 + \frac{1}{2}\chi_1^2$. Moreover, the sampling distribution of the summed ΔG^2 across N independent data sets is given by $\overline{\chi}^2 \sim \sum_{i=0}^{N} \frac{1}{2}^N \binom{N}{i} \chi_i^2$. Note that χ_0^2 is a distribution that concentrates all probability mass on 0. In order to avoid confusion with G^2 values that are evaluated on the basis of traditional χ^2 distributions, we will denote the misfits coming from inequality restrictions with $\Delta\overline{G}^2$.

In order to fully evaluate inequalities such as $r_1 > r_2$, we first test the $r_1 \geq r_2$ inequality restriction against the case in which no restriction is imposed on the two parameters. This is followed by a second test, between the inequality $r_1 \geq r_2$ and the equality restriction $r_1 = r_2$. If the inequality $r_1 > r_2$ represents the most suitable parameter restriction, the result of the first test should fail to reach statistical significance ($p > .05$) but statistically significant results ($p < .05$) should be obtained on the second test. The first test evaluates the magnitude of the misfits produced by the inequality restriction (in comparison to the unrestricted model). The second test, conditional on the non-rejection of the inequality restriction in the first test, evaluates whether the latter can be attributed to parameter equality.

Use of the recognition heuristic

The main goal of our reanalysis was to observe how use of RH is affected by different recognition experiences. The three approaches made distinct predictions about the pattern between the two r parameters. We observed that the probability estimate of using the recognition heuristic is higher in the R^+-U tree in all data sets ($r_1 > r_2$). Therefore, we tested this pattern by first testing the $r_1 \geq r_2$ inequality restriction against the unrestricted model, which did not increase model misfit at all in any of the data sets (all $\Delta\overline{G}^2 = 0, p = 1$). To test for cases of equality, next we tested the equality restriction $r_1 = r_2$, for which increase in model misfit was significant for all data sets (smallest $\Delta\overline{G}^2 = 4.74, p = .01$), except for Data Set 16 ($\Delta\overline{G}^2 = 2.63, p = .05$). The sum of differences in model fit for all data sets was significant ($\Delta\overline{G}^2 = 463.74, p < .01$). The overall pattern of results supports the predictions of the MSH. These results are in line with what we observed with the FIA-based model comparison analysis.

Tendency to choose R^+ while ignoring specific knowledge

The parameter k represents a strategy that can be described as the tendency to choose R^+ over mR while ignoring specific knowledge. The MSH predicts that this strategy will be observed. The presence of this particular strategy across the data sets was tested via the restriction $k = 0$: The restriction led to statistically significant misfits in all single data sets (smallest $\Delta\overline{G}^2 = 8.56$, $p < .01$). In accordance with the MSH prediction, we observe that the proportion of use of this strategy is always unequal to zero. The FIA-based results lead to the same conclusion.

Recognition Validity

We argued that for the MSH to be an ecologically rational strategy, a_1 should be larger than a_2. By analyzing the parameter estimates, it is observable that a_1 is consistently larger than a_2, except for Data Set 4, where the pattern is reversed, and Data Set 14, where they are equal.

To test the significance of such pattern, we first compared the $a_1 \geq a_2$ inequality restriction to the full model. For 15 of the 16 data sets, decrement in model fit was not significant (largest $\Delta\overline{G}^2 = .02, p = .44$). Only in Data Set 4, where the pattern was reversed, there was a significant decrement in model fit ($\Delta\overline{G}^2 = 5.73, p = .01$). The sum of differences in fit of all data sets was not significant ($\Delta\overline{G}^2 = 5.75, p = .65$). To test for cases of equality, next we compared the $a_1 \geq a_2$ inequality restriction with the equality restriction $a_1 = a_2$. The increase in model misfit was significant for 12 of the 16 data sets (smallest $\Delta\overline{G}^2 = 5.09, p < .01$), but not for the remaining four (largest $\Delta\overline{G}^2 = 0.74, p = .19$). The results suggest that in Data Sets 8, 14 and 16 a_1 is equal to a_2, while in Data Set 4, a_1 is either smaller than a_2 or they are equal (for the present analysis, it makes no difference which one is correct). The sum of differences in fit of all data sets was significant ($\Delta\overline{G}^2 = 502.93, p < .01$). The overall pattern fits the predictions of the MSH. These results again match the results from the FIA-based analysis.

References

Iverson, G. J. (2006). An essay on inequalities and order-restricted inference. *Journal of Mathematical Psychology*, *50*(3), 215–219.

Self, S. G., & Liang, K.-Y. (1987). Asymptotic properties of maximum likelihood estimators and likelihood ratio tests under nonstandard conditions. *Journal of the American Statistical Association*, *82*(398), 605–610.

Shapiro, A. (1985). Asymptotic distribution of test statistics in the analysis of moment structures under inequality constraints. *Biometrika*, *72*(1), 133–144.
